# Gender mainstreaming: A lasting solution to disaster risk reduction

**DOI:** 10.4102/jamba.v11i3.723

**Published:** 2019-07-04

**Authors:** Jestina Chineka, Agnes Musyoki, Edmore Kori, Hector Chikoore

**Affiliations:** 1 Department of Geography and Geo-Information Science, University of Venda, Thohoyandou, South Africa

**Keywords:** Disaster Risk Reduction, Gender Mainstreaming, Sustainable Development, Holistic Integration, Overlooking Gender, First Line of Action

## Abstract

Disasters threaten resources as well as displace millions of people globally. It is undisputable that disasters have gender dimensions. However, most African countries are still lagging behind as far as the holistic integration of gender mainstreaming into national policies, particularly on Disaster Risk Reduction (DRR). The purpose of this article was to unpack the consequence of overlooking gender in DRR, thereby highlighting its importance. The study followed a comparative study design, by using two case studies of Mumbwa District in Zambia and Chivi District in Zimbabwe. The specific objectives were to examine the disaster risks in both Mumbwa and Chivi districts, analyse the DRR strategies used in both districts and highlight the critical success factors derived from gender mainstreaming in DRR in these cases. Findings showed that gender mainstreaming is not only an important tool in DRR but also a sustainable development initiative. Despite challenges faced by countries in DRR, gender mainstreaming should always be considered as their first line of action in DRR.

## Introduction

Disaster risks threaten economies and livelihoods in many African countries. According to the International Strategy for Disaster Risk Reduction (ISDR 2004), Africa is the only continent with an increasing disaster trend in the past decade. With climate change effects becoming a reality in the 21st century, a multiplicity of challenges and vulnerabilities which might be having gender connotations have surfaced. Despite the improvements in information technology, education and growing economies, Millennium Development Goals Report (2014) describes multiple and interdependent challenges that have exposed African countries to disaster risks. Recent researches by ISDR (2004), Joseph-Brown and Tuiloma-Sua (2012) and Turnbull, Sterrett and Hilliboe (2013) show that women are affected by disasters more than men, and therefore, women can play a crucial role in disaster management if they are integrated into Disaster Risk Reduction (DRR) plans. International frameworks such as the Hyogo Framework on Disaster Risk Reduction (2005–2015) place gender issues at the centre of disaster management. African countries including Zambia and Zimbabwe are facing a myriad of challenges with regard to different kinds of disasters. According to Chaguta (2010), most African countries demonstrate limited capacity to develop sustainable DRR programmes. The major research question behind this article is: How critical is gender mainstreaming in DRR programmes?

## Study aim and objectives

The aim of this study was to highlight gender mainstreaming as an imperative and holistic, cost-effective and long-term solution to DRR using two cases from Zambia and Zimbabwe. This aim is addressed by the following objectives:

1. to examine the disaster risks in both Mumbwa and Chivi districts
2. to analyse the DRR strategies used in both districts
3. to highlight the critical success factors derived from gender mainstreaming in DRR in these cases.

### The study area

Mumbwa District lies in the western part of the Central Province of Zambia. According to the Central Statistical Office of Zambia (2010), Mumbwa District has a population of 226 177 of which women constituted more than half of the total population. The area has an average annual maximum temperature of 30°C, with mean annual rainfalls ranging between 600 mm and 800 mm (Ministry of Tourism and Arts [Zambia] 2018). As such, drought frequency in Mumbwa District has increased to below the traditionally known 10-year interval (James-Sebro 2005). Drought risks in the Mumbwa District also include reduced agricultural production, water shortages, loss of livestock, malnutrition and poverty (Petrie et al. 2016). To reduce the risks of drought, an international organisation called Heifer International introduced the Pass on the Gift Programme in 2005 in Mumbwa District; the programme’s main objective was to help the community adapt to drought shocks through empowering women. In this programme, women in Mumbwa District were given heifers, which they would pass on the calf to a fellow member in the programme. Livestock is a crucial asset in drought-prone areas. It is a source of the much needed proteins both in meat and milk, as well as being used as draught power by many rural households.

For the second case study, Chivi District located southwest of the Masvingo Province in Zimbabwe was chosen. Chivi District has a population of 166 049, with women forming a majority – 54.5% (ZimSat 2012). Like in Mumbwa District of Zambia, Chivi District of Zimbabwe also experiences drought-induced disasters. The drought frequency in Chivi District lies at an interval of 3–5 years (Mudzonga 2012). Drought risks in Chivi District include dwindling water resources, lack of food and deepening poverty (source). The Government of Zimbabwe through the Ministry of Lands and Agricultural Resources supported by the International Crops Research Institute for the Semi-Arid Tropics (ICRISAT) introduced the Conservation Farming Programme in 2004 to reduce drought shocks through sustainable farming. This programme entailed conservative farming practices such as digging planting basins, mulching and crop rotation. The main objective of this initiative was to reduce the effect of drought by enhancing agricultural productivity.

### Materials and methods

This article is a desktop research based on studies conducted by James-Sebro (2005) and Heifer (2010) in the Mumbwa District of Zambia and studies conducted by Mazvimavi et al. (2010) and Gukurume, Nhodo & Mafongoya (2010) in Chivi District of Zimbabwe. The main themes of these two studies were to build resilient communities through harnessing livestock and to ensure food security in drought-prone rural communities through conservative farming practices. Both cases aimed to reduce the knock-on effects of drought. However, their execution differed in that the Chivi case sought to improve productivity through green agriculture, whereas the Zambian case strengthened communities through empowering women with livestock.

The article follows a comparative research design, a research methodology that aims to make comparisons across different spatial locations, principles or values. Camari (2008) defines it as the juxtaposition of values shared by two or more cases or a method to analyse relationships between phenomena and their casual connections. This design is critical in the contextualisation of the two case areas and in gaining better understanding of the outcomes. The comparative research design helped to highlight the different approaches of the two cases to DRR and the success factors to DRR.

Data analysis was performed by comparing the two cases by using international organisations standards as benchmarks. Strategies from the United Nations International Strategy for Disaster Reduction (UNISDR) such as the Post 1999 Strategy, the Hyogo Framework on DRR (2005) and the Sustainable Livelihoods Framework were employed in this study. This study also employs a theoretical framework from Sen’s Capabilities Theory (1989) to demonstrate the role of women’s capabilities in DRR programmes. Cross patterns from both case studies (Mumbwa and Chivi districts) were examined and interpreted based on the themes generated by the objectives, the analytical framework and case outcomes. The methodological flow adopted starts with case study selection, case outcomes and then interpretation of results.

## Literature review

### Gender mainstreaming concept

Gender mainstreaming is a strategy for gendered concerns and experiences to be integrated into design, policies and programmes in all spheres (World Health Organisation 2010). According to ECOSOC (1997), gender mainstreaming is the process of evaluating gender dimensions of any planned action, legal framework and programme for a specific area at all stages. Tsui, Hearn & Young (2014), defined it as the reorganisation, improvement, development and evaluation of policy processes so that gender equality is incorporated in all policies at all levels and stages. This is conceded by Alston (2014), who described it as a globally accepted strategy for promoting gender equality. They argue that mainstreaming is not an ending solution but a strategy to achieve gender equality by ensuring that it forms the centre of all activities, plans and policies of development.

Sustainable Development Goal number 5 seeks to achieve gender equality and empower all women and girls (UN Women 2015). It targets to end all forms of discrimination and violence against women. It recognises that women have a role to play in their communities, and hence, there is a need to evade gender inequality, especially in DRR.

### Why gender mainstreaming in disaster risk reduction?

Disaster, according to the ISDR (2004), refers to the disruption of the community’s functionality, resulting in human, economic and environmental losses to an extent that the community cannot cope without external support. And risk is defined as the function of a hazard and vulnerability, that is, Risk = Hazard × (Vulnerability ˗ Capacity) (Joseph-Brown 2012). Both women and men face challenges that increase their vulnerability to disaster, in relation to their differentiated social and economic orientation. Turnbull et al. (2013) note that people’s distinct socio-economic clusters shape their disaster experiences. It is within this breadth that it becomes imperative to integrate all gender concerns in disaster management.

Global change has become a fact of life, and disasters are on the increase. Turnbull et al. (2013) highlight that the frequency of disasters is increasing since 1970s globally. IPCC (2007) predicts an increase of 1.1 °C to 6.4 °C in global surface warming by 2100. The increase in global warming means further melting of ice caps in polar regions, rise of sea levels, drier arid regions, floods and droughts. There is a real need to come up with effective strategies in disaster risk reduction.

International organisations recognise the importance of gender mainstreaming if disaster reduction plans are to be sustainable. The United Nations International Strategy for Disaster Reduction (UNISDR) Hyogo Framework for Action (2005–2015) views gender as an integral part in disaster risk management, and hence, it should be incorporated into policies and plans. The Millennium Developmental Goal number 3 places gender equality as one of the vital tools in development.

Women and men continue to have different access to vital resources globally because of cultural and societal values and expectations. In Africa and many Asian countries, most women still lack access to crucial resources which can add their resilience. Musyoki and Khayesi (2012) note that most women lack access to land, livestock and financial capital which are critical in reducing vulnerabilities to disasters. Also, their traditional gender roles of household caretaking reduce their mobility and social networking. Few et al. (2004), citing the Mozambique floods disaster, concede this view and add that women’s confinement to households and caregiving make them often lose their lives to save others.

The rise in disasters globally makes careful planning and a holistic approach to DRR critical. Disasters are now believed to be a manifestation of poor planning and weak policies. According to Turnbull et al. (2013), disasters are an avoidable interruption which requires effective systems and sustainable strategies. They go without say that gender inequality is one of the weak lines of many disaster policies globally.

This draws this article back to the view that drought risks cut across the social fabric and are divided or shaped by age, ethnicity, physical ability and gender. Hence, the socially excluded and economically insecure are worst affected. Gender mainstreaming becomes critical especially in the patriarchal African communities. It goes without saying that effective DRR programmes must be engendered.

## Ethical consideration

This article followed all ethical standards for a research without direct contact with human or animal subjects.

## Results and discussion

In this section, the article highlights the disaster risks experienced, the DRR strategies adopted, as well as the critical success factors derived from gender mainstreaming by using a comparative case analysis of the districts of Mumbwa in Zambia and Chivi in Zimbabwe.

### Disaster risks under case studies

Chivi District despite being a drought prone area, the economy base is centred on agriculture. The small holder farmers in this community often have their livestock and crop production disrupted by the several episodes of drought experienced in the area. Food insecurity is a major issue in the area. According to Chineka (2016), drought exposes the Chivi community across all gender lines. The Household Vulnerability Index weighing vulnerability across physical, social, human, financial and natural assets of each household showed that resilience linked more to economic and social network issues. Mazvimavi et al. (2010) noted that half of the Zimbabwean population are food and nutrition insecure. In response to this risk, several drought adaptation programmes were initiated including conservation farming.

Drought coupled with diseases caused a huge cattle loss in Mumbwa District. According to Heifer (2010), farming became labour-intensive, farming plots were reduced and women abandoned their protein-rich crop fields to work on their men’s staple food crop plots, as men migrated to urban areas seeking employment. Agricultural production dwindled. The community became food and nutrition insecure. Heifer introduced multi-purpose draught cattle programme that could easily be managed by women, in a non-stereotypic approach.

### Chivi and Mumbwa disaster risk reduction strategies

Conservation farming in Chivi targeted to reduce drought shocks through increasing agricultural production. It sought to alleviate drought risk through sustainable green farming. Farmers participated on voluntary basis. They allocated 0.1 hectares of their land for conservation farming. To maximise water and manure, farmers would only plant crops in prepared pits. The programme empowered farmers with sustainable farming practices such as mulching and integrated pest management. According to Gukurume et al. (2010), conservation project brought several conflicts and challenges. Farmers who were participating were shunned by fellow community members who preferred food relief. The project was too labour-intensive. Farmers became reluctant to increase their plots.

Mumbwa adopted the pass on the gift programme. This intervention targeted mostly women, men and youth. A heifer would be passed to a family under the ownership of a woman, and if it produces, they would pass the calf to the next person on the list. The conservation farming programme in Chivi tackled drought from the farmer’s angle, whereas the pass on the gift sought to reduce drought vulnerability through empowering women. This cattle provision project aimed to build economic and environmental resilience through building self-reliant community by increasing crop yields and nutrition as well as promoting agro-ecological farming. This was achieved by building the capacity of rural women and men. Both women and men were trained in rearing cattle. The project targeted social development through gender integration. The project was a huge success, and communities formed their own co-operatives from this project. It spread to other communities and was adopted by other NGOs such as World Vision.

Although both programmes sought to reduce drought risks, they reflect critical gender dimensions. The Chivi case bundled farmers as a homogenous entity. Chivi District has a high female population which constitute most the smallholder farmers. Mumbwa case study brought in gender paradigm. It is also crucial to note that although the Heifer project targeted to empower women, it integrated men as well, which is gender mainstreaming in its holistic sense. Although addressing the past gender inequalities in patriarchal societies is important, the fact is that men still make decision in critical issues and are also vulnerable to disaster shocks, hence the need to harmonise both parties and allow them to showcase their capabilities.

### Critical success factors on disaster risk reduction

Mumbwa District project tackled disaster management from grass-roots level, in which community members participated in every step of the intervention programme. Mumbwa project’s success rests on the inclusive approach more specifically on gender integration. The project ran almost concurrently with gender education, monitoring and reviewing. According to Heifer (2010), the initial step for a group to be co-opted into the programme, it had to have a 50% women membership to avoid repeating of the Women in Development concept. Couples were encouraged to join and contracts of family ownership of livestock were signed to encourage family integration. Women contributing 70% of global disaster victims and gendered asymmetries characterising disaster shocks, gender mainstreaming is the most sustainable tool in DRR (FAO 2007). Although statistics show women domination in subsistence farming, in rural areas such as Chivi, cultural values are still intact; women might be involved in farming, but the land and decision-making remain a male head’s role.

Conservation agriculture being a sound, environmentally friendly approach, it is a fact that it is labour-intensive and adds on to the high demanding gender roles of women. Gender mainstreaming opens up the real needs of the community and results in responsive and sustainable projects formulation. The life span of the conservation agriculture project in Chivi was dependent on donor support. Gukurume et al. (2010) note the 29.3% withdrawal of farmers in 2011–2012 when the donors pulled out. A project falling apart after donors have pulled the plug reflects failure to capacitate communities as well as a lack of project buy-in by its beneficiaries that is common in top–down project designs.

### Lessons from Mumbwa and Chivi

Overlooking gender issues in DRR programmes is not only the overlooking of women and men dilemmas but also a shear negligence of root causes of disaster shocks in the communities. Analysing the social construct of disasters, Enarson (2005) notes that women and men have short- and long-term needs in any disaster. Hence, excluding their participation will be excluding their needs and potentials to adapt to the shocks and capacitate themselves to future disasters. DRR approach in disaster management seeks to promote community resilience through early warning systems and sustainable addressing of long-term crisis. This however to succeed would need disaster shocks to be nipped in the bud, and hence, gender integration is the way to achieve this. Capacitating communities and giving them a say in their issues promotes independence and a sense of proprietorship. In Mumbwa, communities formed a multiple of society projects after the pass on the Gift programme because of interior motivation, although some demo plots for conservation agriculture in Chivi were neglected. Exclusion of beneficiaries does not only result in white elephant projects but also makes the projects unsustainable. According to Sen ’s Capabilities Theory (1989), leaving out a certain gender means leaving out a portion of the society, yet success of any developmental project is depended on people’s capabilities, which vary across the board. Hence, it is not only about gender but community capabilities integration.

Gender is a sensitive topic in rural communities, and hence to address, it would require more subtle approaches to achieve gender balance. Gender awareness training, a continuous assessment and review of gender projects would in the long run build gender equity. Conservation agriculture is eco-friendly and has a potential to boost productivity; however, it is labour-intensive. For people in Chivi to have appreciated it more, the community plot (Munda wezunde raMambo) could have been made the demo plot, and then community work collectively and reap the benefits of it. This would have maintained the traditional practices and strengthen social cohesion against a disaster risk, which is crucial in building resilient communities.

It is vital to note that the success of the Mumbwa District project was not only hinged on gender integration, but also on community capabilities. Most rural programmes fail because of exclusion of some community members. Social cohesion is crucial for any development. In rural communities, societal acceptance matters and so are their perspectives, and hence, residents do not operate in isolation. For DRR programmes like any other community programmes to be sustainable, they have to promote social cohesion, seek to empower the beneficiaries, preserve traditional practices and integrate gender and youth practically. The sustainability concept supports this and highlights that economic development and environmental sustainability cannot be achieved in isolation of the social aspect. In human livelihoods, gender forms the core of the social continuum. Hence to address issues that affect any society, the core must be levelled and rationalised to accept new developments.

## Conclusion

This article concludes that disasters affect communities, and these units are not homogenous. They are underlined by thick social dimensions in which gender plays an integral part, especially in the African context. Results of this study showed that gender mainstreaming was Mumbwa’s secret to the success of its DRR projects. Lacking thereof it in Chivi’s case weakened the Conservation Agriculture Project. Recognition of gender lines does not only address social injustices in communities but also builds social cohesion, thereby strengthening social networks that are vital in DRR. Hence, gender mainstreaming is not only a critical tool in DRR but also a vital step towards sustainable development. Gender mainstreaming must compliment all set of activities in any DRR programme. Governments should get more involved in community programmes by prioritising women’s and men’s capabilities.

This study also recommends that there should not be a disparity between the gender mainstreaming frameworks and communities they are drawn for. This is common with generic frameworks that tend to overlook the subtle social lines binding every community. A top–down approach to a disaster project management is detrimental to disaster risk reduction. Communities need to own a project for them to build an intrinsic motivation towards its success, and only this can yield a sustainable project.

It is undisputable that rural women carry a heavy workload as far as gender roles are concerned. This makes them immobile and lack social networks and ultimately vulnerable when disasters strike. Hence, DRR projects should take cognisance of this – labour-intensive projects such as the conservation farming add an extra burden. Better technologies can be sought to ensure adaptability of these communities to disaster risks. Drought-resistant crop varieties can be researched and be used in the area instead of changing the farming system.

Chivi is a patriarchal community; women manage farms on behalf of their husbands. Hence, major decisions such as changing the farming system will take time to implement as they must be made by men who in most cases work far off the family compound. Moreover, conservation farming practices such as *Dhiga udye* create gender tension. Smallholder farmers in Chivi who are mostly women use mixed farming method; they grow crops and practise livestock ranching. Using the crop residues for mulching instead of storing it for cattle feed use in dry periods will create tension with their spouses who own most of the cattle. Sustainable disaster risk reduction projects seek to integrate gender through sound gender-mainstreamed project designs and implementation plans. Faced with increasing effects of climate change, focus has shifted from adapting to scouting for sustainable adaptation strategies. Hence, continuous monitoring and evaluation of adaptation projects is vital to ensure disaster reduction in communities. Future research can seek effective ways for rural communities to adapt to climate change and its related risks without necessarily disrupting the social systems and creating gender conflicts.
